# The septum transversum mesenchyme induces gall bladder development

**DOI:** 10.1242/bio.20135348

**Published:** 2013-06-20

**Authors:** Yohei Saito, Takuya Kojima, Naoki Takahashi

**Affiliations:** 1 Department of Applied Biological Chemistry, Graduate School of Agricultural and Life Sciences, The University of Tokyo, 1-1-1 Yayoi, Bunkyo-ku, Tokyo 113-8657, Japan; 2 RNA Company Limited, 7-25-7, Nishikamata, Ota-ku, Tokyo 144-8661, Japan

**Keywords:** Septum transversum mesenchyme, Liver, Gall bladder, Ventral pancreas, Mouse

## Abstract

The liver, gall bladder, and ventral pancreas are formed from the posterior region of the ventral foregut. After hepatic induction, *Sox17*+/*Pdx1*+ pancreatobiliary common progenitor cells differentiate into *Sox17*+/*Pdx1−* gall bladder progenitors and *Sox17−/Pdx1*+ ventral pancreatic progenitors, but the cell-extrinsic signals that regulate this differentiation process are unknown. This study shows that the septum transversum mesenchyme (STM) grows in the posterior direction after E8.5, becoming adjacent to the presumptive gall bladder region, to induce gall bladder development. In this induction process, STM-derived BMP4 induces differentiation from common progenitor cells adjacent to the STM into gall bladder progenitor cells, by maintaining *Sox17* expression and suppressing *Pdx1* expression. Furthermore, the STM suppresses ectopic activation of the liver program in the posterior region of the ventral foregut following hepatic induction through an Fgf10/Fgfr2b/Sox9 signaling pathway. Thus, the STM plays pivotal roles in gall bladder development by both inductive and suppressive effects.

## Introduction

The liver, gall bladder, and ventral pancreas emerge from the ventral foregut endoderm during development. Shortly after the liver begins to form, the gall bladder and ventral pancreas form from the remaining ventral foregut endoderm. During organogenesis, various genes expressed in the ventral foregut endoderm play important roles in a cell-autonomous manner. For instance, *Pdx1* (Offield et al., 1996), *Ptf1a* (Kawaguchi et al., 2002; Krapp et al., 1998), *Hhex* (Bort et al., 2004; Hunter et al., 2007), *Hnf6* (Clotman et al., 2002), *Hes1* (Sumazaki et al., 2004), and *Sox17* (Spence et al., 2009; Uemura et al., 2010) are important for the formation of the gall bladder and/or ventral pancreas. *Sox17* functions as a master regulator of the differentiation of ventral foregut endodermal cells into gall bladder progenitor cells. In addition, interconversion between the gall bladder and the ventral pancreas, or between the ventral pancreas and the duodenum occurs in *Sox17*-, *Hes1*-, or *Ptf1a*-deficient mice (Kawaguchi et al., 2002; Spence et al., 2009; Sumazaki et al., 2004), indicating that these genes function cell-autonomously in the region-specific formation of individual organs.

In the absence of the cardiac mesoderm, the isolated mouse ventral foregut endoderm activates the pancreas marker, *Pdx1*, but not the liver marker, *Alb* (Deutsch et al., 2001), illustrating that not only cell-autonomous functions of genes expressed in the ventral foregut endoderm, but also interactions between the ventral foregut endoderm and the adjacent mesenchyme, play important roles in region-specific organogenesis. As mesenchyme-derived factors involved in these interactions, FGF from the cardiac mesoderm (Jung et al., 1999) and BMP from the STM (Rossi et al., 2001) play important roles in hepatic induction. After hepatic induction, pancreatobiliary common progenitor cells (*Sox17*+, *Pdx1*+) segregate into gall bladder progenitor (*Sox17*+, *Pdx1−*) and ventral pancreatic progenitor (*Sox17−*, *Pdx1*+) cells in a *Sox17*-dependent manner (Spence et al., 2009). However, it is unclear which extrinsic signals enable lineage segregation from pancreatobiliary common progenitor cells by regulating *Sox17* and *Pdx1* expression.

Pancreatic induction occurs in the region where FGF is either low or absent (Deutsch et al., 2001), that is, in the region separated from the cardiac mesoderm that expresses *Fgf*. In *Hhex*-null embryos, pancreas formation does not occur in the ventral foregut unless the ventral foregut is isolated from the adjacent mesenchyme (Bort et al., 2004). This is possibly because the nascent pancreatic region is not well separated from the cardiac mesoderm in *Hhex*-null embryos due to elongation defects in the ventral foregut resulting from decreased cell proliferation. These results indicate that organogenesis in the ventral foregut endoderm is induced when the appropriate region of the ventral foregut endoderm receives mesenchyme-derived signals at the proper time.

The STM originates from the lateral plate mesoderm (Douarin, 1975; Fukuda-Taira, 1981; Sherer, 1975) and is adjacent to the ventral foregut endoderm during ventral foregut-derived organogenesis. *Gata4* (Watt et al., 2004) and *Mab21l2* (Saito et al., 2012) are essential for STM formation. *Gata4* and *Mab21l2* are expressed in the STM, and in *Gata4*- and *Mab21l2*-deficient embryos, defective morphogenesis of the STM occurs. The STM is involved not only in hepatic induction, but also in the growth and survival of hepatoblasts, which are bipotential progenitors for hepatocytes and cholangiocytes (Zaret, 2002), demonstrating that the STM is important for liver development. However, it is not clear from previous studies using *Gata4*- or *Mab21l2*-deficient mice what roles the STM plays during gall bladder and ventral pancreas development. Therefore, the aim of this study was to determine at which point during development the STM is adjacent to the nascent gall bladder and/or ventral pancreas in the ventral foregut, and how the STM is involved in the development of these organs using *Mab21l2*-null embryos as a model for STM loss.

In this study, we show that the STM becomes adjacent to the nascent gall bladder region after embryonic day 9.0 (E9.0). This cellular event triggers the differentiation of pancreatobiliary common progenitor cells into gall bladder progenitor cells by maintaining *Sox17* expression and suppressing *Pdx1* expression. Moreover, after hepatic induction, STM-derived signals act on the nascent gall bladder and ventral pancreas regions to suppress ectopic induction of the liver program. Taken together, these observations indicate that following hepatic induction, the STM induces gall bladder development, while suppressing ectopic activation of the liver program in the posterior region of the ventral foregut.

## Materials and Methods

### Mice

The generation of the mutant mice used in this study has been reported previously (Yamada et al., 2004). Mice were backcrossed to the ICR strain. Knockout embryos are more readily obtained via the interbreeding of heterozygous mice using the ICR strain, because the litter size of the ICR strain is larger than that of the B6 strain. There are no differences in phenotype or lethality rates at each developmental stage between the ICR and B6 strains. Mice were maintained in accordance with protocols approved by the Animal Care and Use Committee of the University of Tokyo.

### *In situ* hybridization

Whole-mount *in situ* hybridization was performed as described previously (Nieto et al., 1996) at 65°C in 50% formamide containing 5×SSC. Paraffin sections were hybridized *in situ* at 65°C in 50% formamide, 20 mM Tris-HCl (pH 8.0), 300 mM NaCl, 0.2% Sarkosyl, 1×Denhart's solution, 10% dextran sulfate, and 0.5 mg/mL yeast tRNA. All probes were labeled with digoxigenin using standard procedures. Details for probes will be provided upon request.

### Histology

Embryos were dissected in phosphate-buffered saline (PBS) and fixed in 4% paraformaldehyde in PBS overnight at 4°C. Fixed embryos were dehydrated through a graded alcohol series and embedded in paraffin, sectioned (8 µm thick), and stained with hematoxylin and eosin.

### Detection of proliferating or apoptotic cells

Paraffin sections were deparaffinized with xylene and dehydrated through a graded ethanol series. Sections were boiled in 10 mM citrate buffer (pH 6.0) for 10 min and washed with PBS. Endogenous peroxidase activity was blocked with 3% hydrogen peroxide in TBS for 10 min. After washing with PBS, sections were incubated with 1/400 anti-PHH3 antibody (CST) overnight. After washing with PBS, sections were incubated with HRP-conjugated goat anti-rabbit IgG and 1% BSA in PBS for 1 hour. Immunoreactive sites were visualized with DAB and H_2_O_2_. Sections were counterstained with hematoxylin.

TdT-mediated dUTP nick-end labeling (TUNEL) analysis was performed as follows. Paraffin sections (8 µm thick) of embryos were incubated in 3% H_2_O_2_ for 15 min, Proteinase K solution for 10 min, and then TdT reaction solution (0.2 mM fluorescein-12-dUTP (Roche), 0.2 mM dATP, 1 mM CoCl_2_, 30 mM Tris-HCl (pH 7.5), 140 mM sodium cacodylate, and 40 U terminal deoxynucleotidyl transferase (Roche)). Reactive sites were detected with an alkaline phosphatase-conjugated anti-fluorescein antibody (Roche) in a solution containing a phosphatase substrate (Fast Red Tablets, Roche). Sections were counterstained with hematoxylin.

### Whole-embryo culture

E9.0 embryos were dissected from the uteri for *in vitro* culture. All littermate embryos were then cultured for 6 hours in medium (DMEM, Gibco) containing 4 nM Noggin (Peprotech), 20 nM BMP4 (R&D Systems), and BSA at 37°C in the presence of 5% CO_2_. Thereafter, embryos were fixed in 4% paraformaldehyde.

## Results

### After E9.0, the STM is adjacent to the *Sox17*, but not the *Pdx1*, expressing region of the foregut

**Fig. 1.**
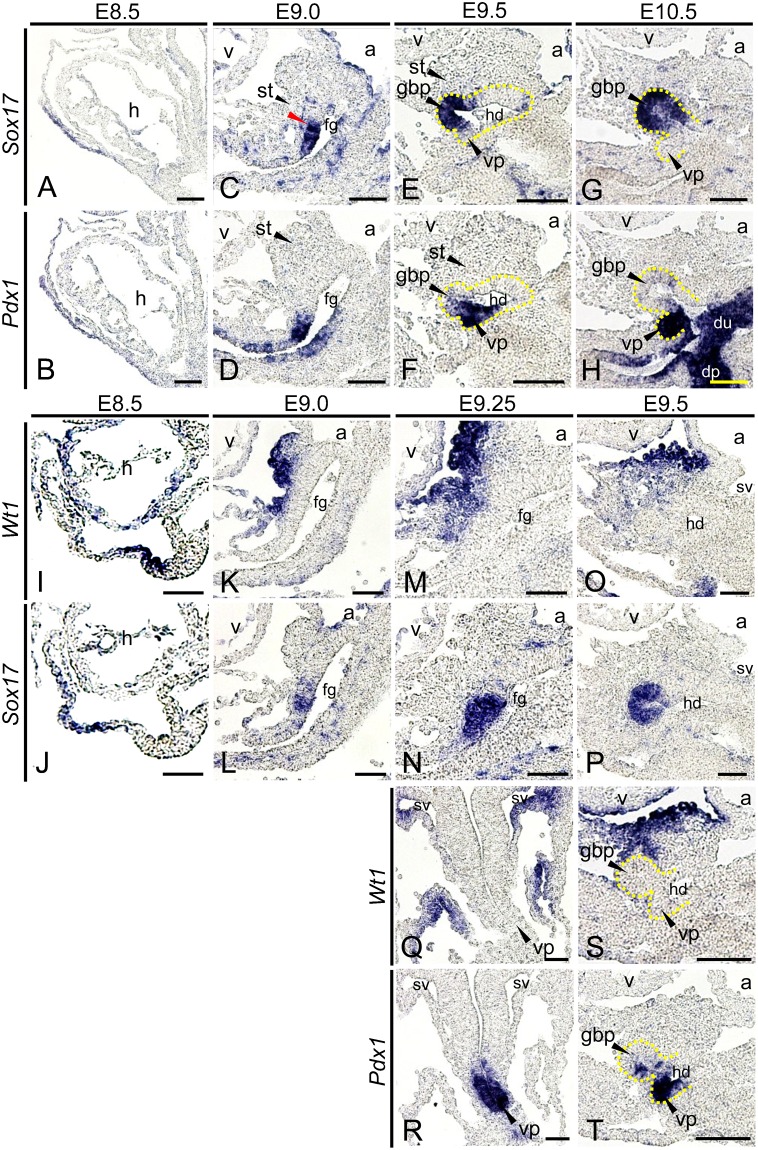
The STM is positioned adjacent to the *Sox17+* region after E9.0. *In situ* hybridization for the indicated transcripts was conducted using paraffin sections of wild-type embryos. (**A–D**) Sagittal serial sections showing that at E8.5 (A,B), *Sox17* and *Pdx1* were expressed in the same region in the nascent foregut, and at E9.0 (C,D), *Sox17*+/*Pdx1−* endodermal cells start to emerge in the ventral foregut (red arrowhead). (**E–H**) Sagittal serial sections showing that at E9.5 (E,F), *Sox17* expression was detected in the gall bladder primordium and the anterior portion of the ventral pancreatic bud, and *Pdx1* expression was detected in the ventral pancreatic bud and the posterior portion of the gall bladder primordium. At E10.5 (G,H), the expression of *Sox17* and *Pdx1* was specifically detected in the gall bladder primordium and the ventral pancreatic bud, respectively. (**I–P**) Sagittal serial sections showing that at E8.5 (I,J), *Sox17* was not expressed in the nascent foregut adjacent to the *Wt1*-expressing region. At E9.0 (K,L), *Sox17* began to be expressed in the ventral foregut adjacent to the *Wt1*-expressing region. At E9.25 (M,N), *Sox17* was expressed in the ventral foregut adjacent to the *Wt1*-expressing region, and at E9.5 (O,P), the *Sox17*-expressing gall bladder primordium was surrounded by the *Wt1*-expressing region. (**Q–T**) Transverse serial sections of an E9.25 embryo (Q,R) showing that *Wt1* was not expressed around the *Pdx1*-expressing ventral pancreatic bud. Sagittal serial sections of an E9.5 embryo (S,T) showing that the *Pdx1*-expressing ventral pancreatic bud was not adjacent to the *Wt1*-expressing region. h, heart; a, atrium; v, ventricle; st, septum transversum mesenchyme; hd, hepatic diverticulum; du, duodenum; dp, dorsal pancreatic bud; vp, ventral pancreatic bud; fg, foregut; sv, sinus venosus; gbp, gall bladder primordium. Scale bars: 30 µm (A–D, I–T), 50 µm (E–H).

During gall bladder and ventral pancreas development, pancreatobiliary common progenitor cells expressing both *Sox17* and *Pdx1* differentiate into *Sox17+/Pdx1−* gall bladder progenitor cells, and *Sox17−/Pdx1+* ventral pancreatic progenitor cells (Spence et al., 2009). However, how this differentiation process is regulated is unknown. Interactions between the endodermal epithelium and adjacent mesenchyme are important during ventral foregut-derived organ development, and mesenchyme-derived signals regulate organ induction in the ventral foregut (Deutsch et al., 2001; Jung et al., 1999; Rossi et al., 2001). Therefore, we examined the relationship between the expression of *Sox17* and *Pdx1* and the position of the adjacent mesenchyme. At E8.5, pancreatobiliary common progenitor cells expressed both *Sox17* and *Pdx1* (Fig. 1A,B) (Spence et al., 2009); *Sox17*+/*Pdx1−* ventral foregut endodermal cells emerged at E9.0 (Fig. 1C,D; red arrowhead). At E9.5, *Sox17* was expressed in the gall bladder primordium and the anterior region of the ventral pancreatic bud, and *Pdx1* was expressed in the ventral pancreatic bud and the posterior region of the gall bladder primordium (Fig. 1E,F). At E10.5, *Sox17* and *Pdx1* were specifically expressed in the gall bladder primordium and in the ventral pancreatic bud, respectively (Fig. 1G,H). These results suggest that after E9.0, the expression of *Sox17* and *Pdx1* is differentially regulated in the posterior region of the ventral foregut, resulting in the tissue-specific separation of expression of these two factors between E9.5 and E10.5. Following hepatic induction from E8.0 to E8.5, the STM is adjacent to the ventral foregut endoderm (Rossi et al., 2001). We examined the positional relationship between the *Sox17+* and *Pdx1+* region and the STM. At E8.5, the region expressing *Wt1*, an STM marker, was separated from the *Sox17*-expressing region (Fig. 1I,J), suggesting that the STM is not adjacent to pancreatobiliary common progenitor cells, that is, the *Sox17*-expressing region. At E9.0, the *Wt1*-expressing region became juxtaposed to the *Sox17*-expressing region (Fig. 1K,L). At E9.25 and E9.5, *Wt1* was expressed adjacent to the *Sox17*-expressing region (Fig. 1M–P), while *Wt1* was not expressed near the *Pdx1*-expressing region (Fig. 1Q–T). These results show that after E9.0, the STM becomes adjacent to the *Sox17*-expressing region, but not the *Pdx1*-expressing region.

### In *Mab21l2*-deficient embryos, complete loss of the STM occurs near the posterior region of the ventral foregut after E9.0

**Fig. 2.**
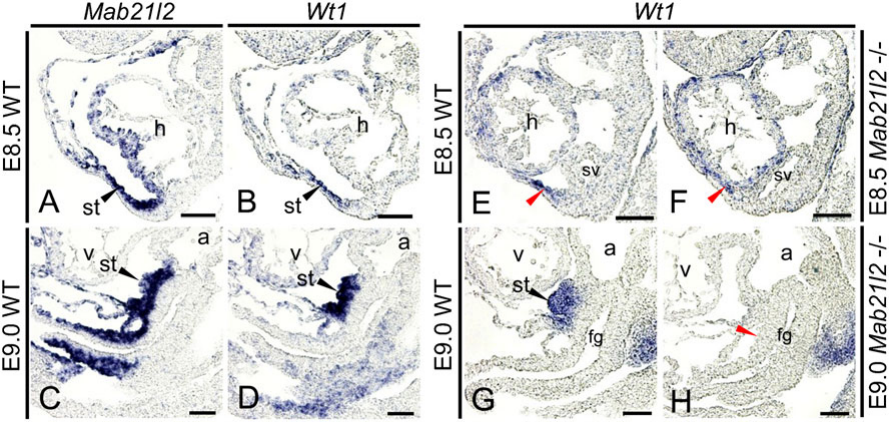
*Mab21l2*-deficient embryos exhibit defective morphogenesis of the STM after E9.0. *In situ* hybridization for the indicated transcripts was conducted using paraffin sections of wild-type and *Mab21l2* mutant embryos. (**A–D**) Sagittal serial sections showing that at E8.5 (A,B), *Mab21l2* was expressed in the *Wt1*-expressing STM near the nascent foregut, and at E9.0 (C,D), *Mab21l2* was expressed in the *Wt1*-expressing STM near the ventral foregut. (**E–H**) Sagittal sections of wild-type and *Mab21l2*-deficient embryos showing that at E8.5 (E,F), *Wt1* expression was detected near the nascent foregut in *Mab21l2*-deficient embryos, similarly to in wild-type embryos (red arrowheads). At E9.0 (G,H), *Wt1* expression was detected in the STM near the ventral foregut in wild-type embryos, but not in *Mab21l2*-deficient embryos (red arrowhead). h, heart; a, atrium; v, ventricle; st, septum transversum mesenchyme; fg, foregut; sv, sinus venosus. Scale bars: 30 µm.

We have shown that *Mab21l2* is expressed in the STM after E8.5. In E9.5 *Mab21l2*-deficient embryos, morphogenesis of the STM was defective, resulting in defective morphogenesis of the liver (Saito et al., 2012). However, it is unclear when defects in STM formation occur. We first examined *Mab21l2* expression in the STM before E9.5. *Mab21l2* was expressed in the *Wt1*-expressing region from E8.5 to E9.0 (Fig. 2A–D), showing that *Mab21l2* is expressed in the STM after E8.5 and is involved in STM formation. We next examined the time at which defective morphogenesis of the STM occurred. At E8.5, *Wt1* expression was detected near the nascent foregut in *Mab21l2*-deficient and wild-type embryos (Fig. 2E,F; red arrowheads), demonstrating that STM formation is normal at E8.5. At E9.0, expression of *Wt1* was detected near the posterior region of the ventral foregut in wild-type embryos, but not in *Mab21l2*-deficient embryos (Fig. 2G,H; red arrowhead), showing that a complete loss of the STM occurs near the posterior region of the ventral foregut in *Mab21l2*-deficient embryos after E9.0. Near the ventral foregut, the STM and endothelial cells were present. At E9.5, *Flk1*, an endothelial cell marker, was expressed adjacent to the gall bladder primordium and the ventral pancreatic bud, indicating that endothelial cells are present next to these regions (supplementary material Fig. S1A). Moreover, *Flk1*-positive endothelial cells appeared normal in *Mab21l2*-deficient embryos compared to wild-type embryos at E9.0 (supplementary material Fig. S1B,C), indicating that the STM is only lost near the posterior region of the ventral foregut in *Mab21l2*-deficient embryos. Therefore, we examined the function of the STM in organogenesis in the posterior region of the ventral foregut after E9.0 using *Mab21l2*-deficient embryos as a model of STM loss.

### Defective morphogenesis of the extrahepatic biliary system and gall bladder occurs in *Mab21l2*-deficient embryos

**Fig. 3.**
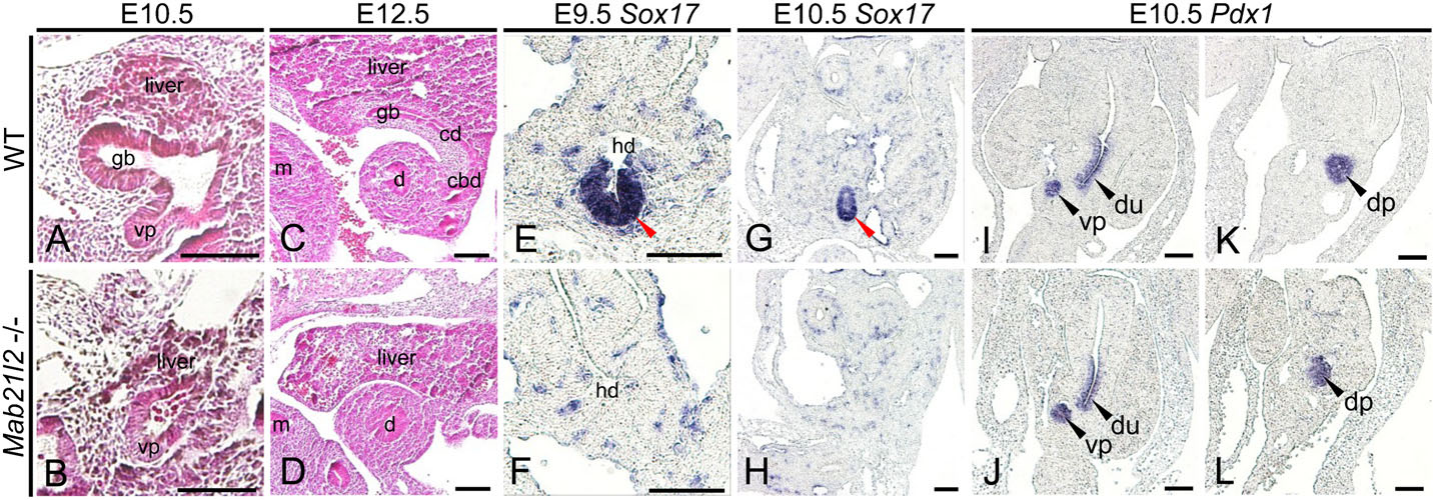
The extrahepatic biliary system, including the gall bladder, is not formed in *Mab21l2*-deficient embryos. (**A–D**) Hematoxylin and eosin (H&E)-stained paraffin sections. (**E–L**) *In situ* hybridizations of paraffin sections. (A–D) Sagittal sections of wild-type and *Mab21l2*-deficient embryos at E10.5 (A,B), showing the gall bladder primordium and ventral pancreatic bud in wild-type embryos; the gall bladder primordium was lost in *Mab21l2*-deficient embryos. At E12.5 (C,D), the extrahepatic biliary system, including the gall bladder, and cystic and common bile ducts, was observed in wild-type embryos, but not in *Mab21l2*-deficient embryos. (E–H) Transverse sections of wild-type and *Mab21l2*-deficient embryos showing that at E9.5 (E,F), the *Sox17*-positive gall bladder primordium emerged from the hepatic diverticulum in wild-type embryos (red arrowhead), but not in *Mab21l2*-deficient embryos. At E10.5 (G,H), the *Sox17*-positive gall bladder primordium was observed in wild-type embryos (red arrowhead), but not in *Mab21l2*-deficient embryos. (I–L) Transverse sections of E10.5 wild-type and *Mab21l2*-deficient embryos showing that *Pdx1+* ventral and dorsal pancreatic buds were observed in *Mab21l2*-deficient embryos, similarly to in wild-type embryos. gb, gall bladder primordium; vp, ventral pancreatic bud; m, midgut; d and du, duodenum; cd, cystic duct; cbd, common bile duct; hd, hepatic diverticulum; dp, dorsal pancreatic bud. Scale bars: 50 µm.

If the STM influences the differentiation of the pancreatobiliary common progenitor cells, then STM loss should affect the formation of the gall bladder and/or ventral pancreas. We examined the formation of the gall bladder and ventral pancreas in *Mab21l2*-deficient embryos. At E10.5, the formation of the liver, gall bladder, and ventral pancreas was detected morphologically in wild-type embryos (Fig. 3A). By contrast, the gall bladder primordium was absent in *Mab21l2*-deficient embryos, although a small liver and normal ventral pancreatic bud were observed (Fig. 3B). At E12.5, the formation of the extrahepatic biliary system, including the gall bladder, was observed in wild-type embryos (Fig. 3C), but not in *Mab21l2*-deficient embryos (Fig. 3D). We next examined gall bladder and pancreas formation in *Mab21l2*-deficient embryos using *Sox17* as a marker of the gall bladder and *Pdx1* as a marker of the pancreas. At E9.5, a *Sox17+* gall bladder primordium arose from the hepatic diverticulum in wild-type embryos (Fig. 3E; red arrowhead), but not in *Mab21l2*-deficient embryos (Fig. 3F). Similarly, at E10.5, the *Sox17+* gall bladder primordium was observed in wild-type embryos (Fig. 3G; red arrowhead), but not in *Mab21l2*-deficient embryos (Fig. 3H). Moreover, the formation of ventral and dorsal pancreas was normal in *Mab21l2*-deficient embryos at E10.5 (Fig. 3J,L), similar to wild-type embryos (Fig. 3I,K). These results reveal that the STM is required for formation of the gall bladder, but not the pancreas.

### Reduced expression of *Sox17* and ectopic expression of *Pdx1* occur in *Mab21l2*-deficient embryos

**Fig. 4.**
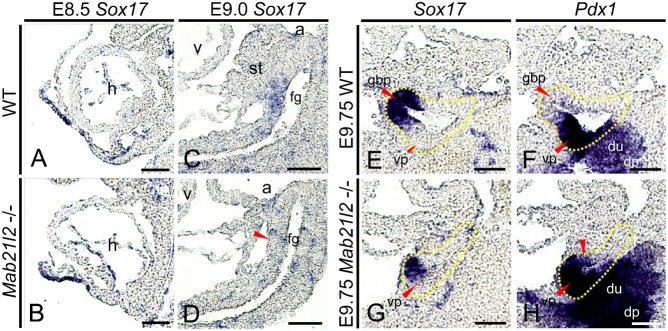
Defects in *Sox17* and *Pdx1* expression occur in *Mab21l2*-deficient embryos. *In situ* hybridization for the indicated transcripts was conducted using paraffin sections of embryos. (**A–D**) Sagittal sections of wild-type and *Mab21l2*-deficient embryos showing that at E8.5 (A,B), *Sox17* expression was detected in the nascent foregut in *Mab21l2*-deficient embryos, similarly to in wild-type embryos. At E9.0 (C,D), *Sox17* was expressed in the ventral foregut in wild-type embryos, but *Sox17* expression was significantly reduced in the ventral foregut in *Mab21l2*-deficient embryos (red arrowhead). (**E–H**) Sagittal serial sections of an E9.75 embryo showing that in wild-type embryos (E,F), *Sox17* was specifically expressed in the gall bladder primordium, and *Pdx1* in the ventral pancreatic bud; in *Mab21l2*-deficient embryos (G,H), *Sox17* was only expressed in the anterior portion of the ventral pancreatic bud, and *Pdx1* was expressed not only in the ventral pancreatic bud, but also in the region where *Sox17* was intrinsically expressed (red arrowhead). a, atrium; v, ventricle; fg, foregut; h, heart; st, septum transversum mesenchyme; gbp, gall bladder primordium; vp, ventral pancreatic bud; du, duodenum; dp, dorsal pancreatic bud. Scale bars: 30 µm (A–D), 50 µm (E–H).

Loss of the gall bladder in *Mab21l2*-deficient embryos suggested that defects occurred during the differentiation of pancreatobiliary common progenitor cells into gall bladder progenitor cells. As *Sox17* plays an essential role in this process (Spence et al., 2009), we examined the expression of *Sox17* in the ventral foregut. At E8.5, the expression of *Sox17* in wild-type embryos (Fig. 4A) was indistinguishable from that in *Mab21l2*-deficient embryos (Fig. 4B). At E9.0, the expression of *Sox17* was significantly reduced in the ventral foregut in *Mab21l2*-deficient embryos (Fig. 4D; red arrowhead) compared to wild-type embryos (Fig. 4C). To examine whether reduced expression of *Sox17* resulted from decreased expression of *Sox17* in the ventral foregut endodermal cells, or from decreased proliferation and/or increased apoptosis of *Sox17+* endodermal cells, proliferating and apoptotic cells in the ventral foregut were examined by immunohistochemistry using the mitosis marker phospho-histone H3 (PHH3) and a terminal deoxynucleotidyl transferase-mediated dUTP nick-end labeling (TUNEL) assay, respectively. At E9.0, there were no differences in PHH3-positive nuclei in the ventral foregut between wild-type (supplementary material Fig. S2A,C) and *Mab21l2*-deficient embryos (supplementary material Fig. S2B,C), indicating that the proliferation of ventral foregut endodermal cells was normal in *Mab21l2*-deficient embryos. Similarly, the number of TUNEL-positive cells was unchanged in the ventral foregut in *Mab21l2*-deficient and wild-type embryos at E9.0 (data not shown). Moreover, elongation defects in the ventral foregut were not detected in *Mab21l2*-deficient embryos (Fig. 2H, Fig. 4D) compared to wild-type embryos (Fig. 2G, Fig. 4C) at E9.0, indicating that the reduced expression of *Sox17* in the ventral foregut resulted from the reduced expression of *Sox17* in endodermal cells, and that the STM is required for the maintenance of *Sox17* in the ventral foregut. At E9.25, the expression of *Pdx1* was unchanged in the ventral foregut in *Mab21l2*-deficient embryos (supplementary material Fig. S3B) compared to wild-type embryos (supplementary material Fig. S3A), demonstrating that the STM is not required for the maintenance of *Pdx1* in the ventral foregut. We next examined the specificity of the *Sox17-* and *Pdx1*-expressing regions at E9.75 when *Sox17* and *Pdx1* are expressed in the gall bladder primordium and ventral pancreatic bud, respectively. In *Mab21l2*-deficient embryos, *Sox17* was only expressed in the anterior region of the ventral pancreatic bud (Fig. 4G). *Pdx1* was expressed in the ventral pancreatic bud and in the region where *Sox17* was intrinsically expressed (Fig. 4H; red arrowhead) compared to wild-type embryos (Fig. 4E,F), showing that the STM is required for the suppression of *Pdx1* in the presumptive gall bladder region. We next examined the expression of genes with important roles in gall bladder formation, which were also expressed in the ventral foregut endoderm. At E9.25, the expression of *Hhex*, *Hnf6*, and *Hes1* in *Mab21l2*-deficient embryos was indistinguishable from that in wild-type embryos (supplementary material Fig. S3C–H). These results illustrate that gall bladder loss resulted specifically from defects in *Sox17* and *Pdx1* expression.

### BMP4 influences the expression of *Sox17* and *Pdx1*

**Fig. 5.**
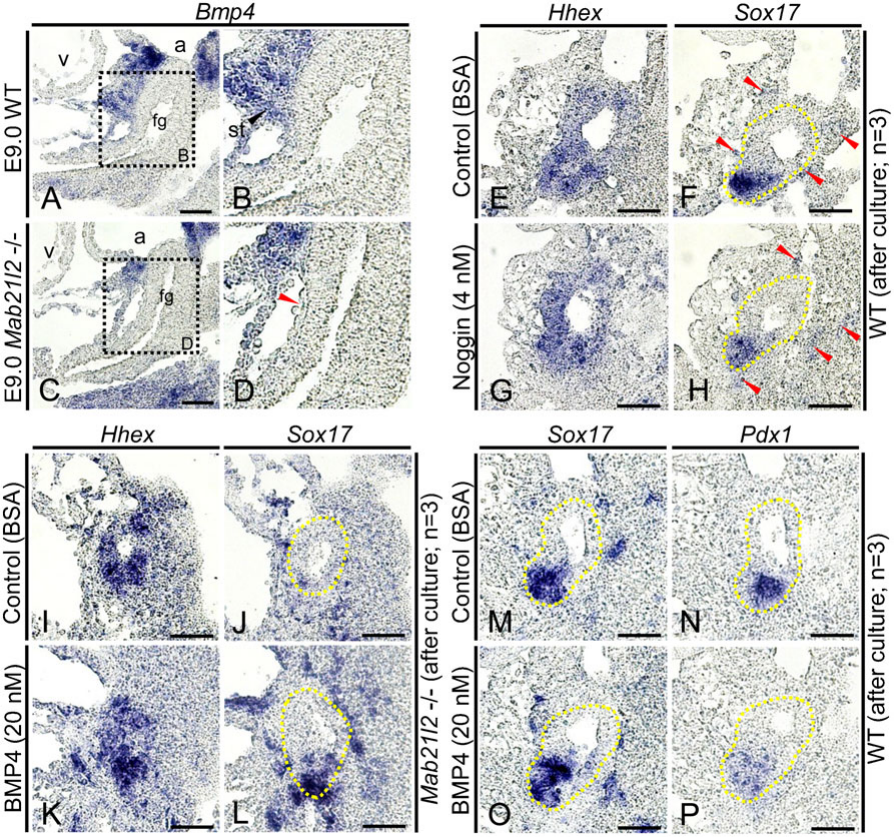
BMP4 influences the regulation of *Sox17* and *Pdx1*. *In situ* hybridization for the indicated transcripts was conducted using embryo paraffin sections. (**A–D**) Sagittal sections of E9.0 wild-type and *Mab21l2*-deficient embryos showing that *Bmp4* expression was detected in the STM in wild-type embryos, but was not expressed near the ventral foregut in *Mab21l2*-deficient embryos (red arrowhead). (**E–P**) Embryo culture experiments were conducted by dissecting E9.0 embryos and culturing them in medium containing Noggin, BMP4, or BSA for 6 hours. After fixation, *Sox17* and *Pdx1* expression was assessed. The results shown here are representative of three independent experiments. (E–H) Sagittal serial sections of wild-type embryos cultured for 6 hours in medium containing Noggin or BSA showing that Noggin had a tendency to reduce the expression of *Sox17* in the *Hhex*-positive foregut region (yellow dashed line) compared to embryos cultured in control medium containing BSA. (I–L) Sagittal serial sections of *Mab21l2*-deficient embryos cultured for 6 hours in medium containing BMP4 or BSA showing that BMP4 had a tendency to rescue *Sox17* expression in the *Hhex*-positive foregut region (yellow dashed line) compared to embryos cultured in control medium. (M–P) Sagittal serial sections of wild-type embryos cultured for 6 hours in medium containing BMP4 or BSA showing that BMP4 had a tendency to increase *Sox17* expression moderately and to reduce *Pdx1* expression in the foregut region (yellow dashed line) compared to embryos cultured in control medium. a, atrium; v, ventricle; fg, foregut. Scale bars: 30 µm.

Our results suggest that STM-derived signals regulate *Sox17* and *Pdx1* expression in the ventral foregut during differentiation of pancreatobiliary common progenitor cells. However, the cell-extrinsic signals that regulate *Sox17* and *Pdx1* during this differentiation process are unknown. Various genes that encode secreted ligands such as BMP, FGF, and HGF are expressed in the STM (Berg et al., 2007; Rossi et al., 2001; Schmidt et al., 1995). However, which of these genes are involved in the regulation of *Sox17* and *Pdx1* expression during gall bladder and/or ventral pancreas development is not clear. *FoxF1*, which encodes a forkhead transcription factor, is expressed in the STM and plays an important role in gall bladder formation because haploinsufficiency of *FoxF1* resulted in defects in gall bladder development (Kalinichenko et al., 2002). Furthermore, previous studies have shown that expression of *Bmp4* is regulated by *FoxF1* (Mahlapuu et al., 2001), and that the BMP receptors BMPRIA, BMPRII, and ActRIIA are expressed in the ventral foregut endoderm (Mishina et al., 1995; Roelen et al., 1997). At E9.0, *Bmp4* was expressed in the STM in wild-type embryos (Fig. 5A,B), but the expression of *Bmp4* was not detected near the posterior region of the ventral foregut in *Mab21l2*-deficient embryos (Fig. 5C,D; red arrowhead), suggesting the possibility that STM-derived BMP4 may regulate the expression of *Sox17* and *Pdx1* in the ventral foregut endoderm. To test this hypothesis, we performed several experiments using whole-embryo cultures. We cultured E9.0 embryos for 6 hours in medium containing 4 nM Noggin, a BMP antagonist, and evaluated its effects on *Sox17* and *Pdx1* expression. Noggin had a tendency to reduce the expression of *Sox17* in the *Hhex*-positive foregut region (*n* = 3; Fig. 5G,H) compared to embryos cultured in control medium containing BSA (Fig. 5E,F). The expression of *Sox17* in endothelial cells around the ventral foregut (red arrowheads) and the size of the *Sox17*-expressing region were the same in control and Noggin-treated embryos. These results indicate that BMPs are involved in the regulation of *Sox17* in the ventral foregut endodermal cells. Noggin did not influence *Pdx1* expression (data not shown). We next cultured E9.0 *Mab21l2*-deficient embryos in medium containing 20 nM BMP4 for 6 hours, to determine whether the expression of *Sox17* could be rescued by BMP4. In *Mab21l2*-deficient embryos cultured in control medium containing BSA, the expression of *Sox17* was significantly reduced in the *Hhex*-positive foregut region (Fig. 5I,J). By contrast, in *Mab21l2*-deficient embryos cultured in medium containing BMP4, *Sox17* expression had a tendency to be rescued in the *Hhex*-positive foregut region (*n* = 3; Fig. 5K,L) and was similar to wild-type embryos cultured in control medium (Fig. 5F). These results suggest that BMP4 is involved in the maintenance of *Sox17* in the ventral foregut. In E9.0 wild-type embryos cultured in medium containing 20 nM BMP4 for 6 hours, BMP4 had a tendency to increase *Sox17* expression moderately (Fig. 5O) and to reduce *Pdx1* expression (Fig. 5P) compared with embryos cultured in control medium (*n* = 3; Fig. 5M,N). These results demonstrate that BMP4 maintains *Sox17* expression and suppresses *Pdx1* expression in the ventral foregut.

### STM loss results in ectopic *Alb* expression and defects in the Fgf10/Fgfr2b/Sox9 signaling pathway in the posterior region of the ventral foregut

**Fig. 6.**
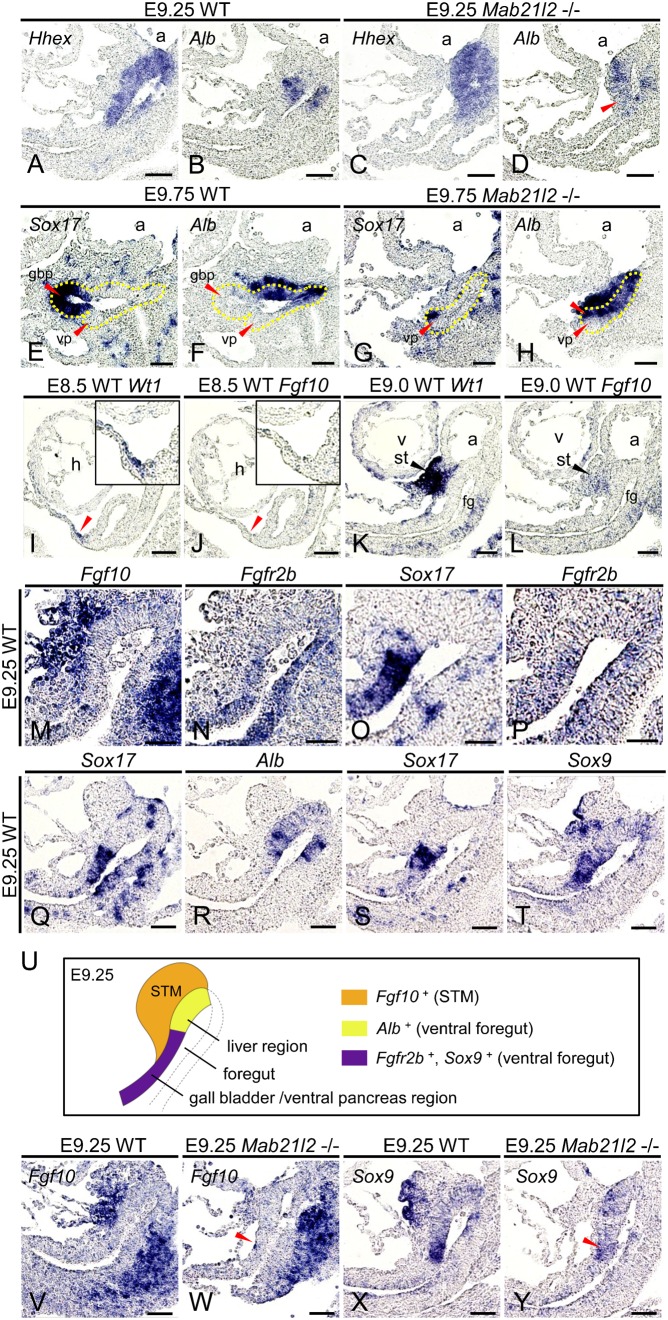
Ectopic *Alb* expression and defects in the Fgf10/Fgfr2b/Sox9 signaling pathway occur in *Mab21l2*-deficient embryos. (**A–T**,**V–Y**) *In situ* hybridization for the indicated transcripts was conducted using paraffin sections of embryos. (A–D) Sagittal serial sections of E9.25 wild-type and *Mab21l2*-deficient embryos showing that *Alb* was expressed only in the anterior portion of the *Hhex*-positive ventral foregut in wild-type embryos, but was expressed in both the anterior and posterior regions of the *Hhex*-positive ventral foregut in *Mab21l2*-deficient embryos (red arrowhead). (E–H) Sagittal serial sections of E9.75 embryos showing that *Sox17* was expressed in the gall bladder primordium and *Alb* was expressed in the nascent liver in wild-type embryos (E,F), but *Sox17* was expressed only in the anterior portion of the ventral pancreatic bud, and *Alb* was expressed not only in the nascent liver, but also in the posterior region of the ventral foregut, including the *Sox17+* region of the ventral pancreatic bud in *Mab21l2*-deficient embryos (red arrowhead) (G,H). (I–L) Sagittal serial sections showing that at E8.5 (I, J), *Fgf10* expression was not detected in the *Wt1*-positive STM (red arrowhead). At E9.0 (K,L), *Fgf10* began to be expressed in the *Wt1*-positive STM. (M,N) Sagittal serial sections of an E9.25 embryo showing that *Fgf10* was expressed in the STM near the ventral foregut and that *Fgfr2b* was expressed in the ventral foregut. (O,P) Sagittal serial sections of an E9.25 embryo showing that both *Fgfr2b* and *Sox17* were expressed in the ventral foregut. (Q,R) Sagittal serial sections of an E9.25 embryo showing that *Sox17* was expressed in the presumptive gall bladder and ventral pancreas, but not in the *Alb+* hepatic region. (S,T) Sagittal serial sections of an E9.25 embryo showing that *Sox9* was mainly expressed in the *Sox17*-expressing ventral foregut. (**U**) Diagrammatic summary of *Fgf10*, *Fgfr2b*, and *Sox9* expression in the ventral foregut in an E9.25 embryo. *Fgf10* is expressed in the STM, whereas *Fgfr2b* and *Sox9* are expressed in the presumptive gall bladder and ventral pancreas, and not in the *Alb*-positive nascent liver. (V,W) Sagittal sections of E9.25 wild-type and *Mab21l2*-deficient embryos showing that *Fgf10* was expressed in the STM near the ventral foregut in wild-type embryos, but *Fgf10* expression was lost in *Mab21l2*-deficient embryos (red arrowhead). (X,Y) Sagittal sections of E9.25 wild-type and *Mab21l2*-deficient embryos showing that *Sox9* was expressed in the ventral foregut in wild-type embryos, but *Sox9* expression was significantly reduced in *Mab21l2*-deficient embryos (red arrowhead). a, atrium; gbp, gall bladder primordium; vp, ventral pancreatic bud. Scale bars: 50 µm (A–T,V–Y).

Previous studies have shown that FGF from the cardiac mesoderm and BMP from the STM induce liver development (Jung et al., 1999; Rossi et al., 2001), while hepatic induction is suppressed in the region where FGF is either low or absent (Deutsch et al., 2001). However, the role of the STM in suppressing liver development is not known, and we therefore examined the function of the STM in the posterior region of the ventral foregut. At E9.25, the expression of *Alb* was only detected in the anterior region of the *Hhex*-positive ventral foregut (i.e., the presumptive liver region) in wild-type embryos (Fig. 6A,B). By contrast, the expression of *Alb* in the *Hhex*-positive ventral foregut was detected not only in the anterior region but also in the posterior region (i.e., the presumptive gall bladder and ventral pancreas region) in *Mab21l2*-deficient embryos (Fig. 6C,D; red arrowhead). Moreover, in E9.75 *Mab21l2*-deficient embryos, *Sox17* was only expressed in part of the anterior region of the ventral pancreatic bud (Fig. 6G). *Alb* was expressed not only in the nascent liver, as in wild-type embryos (Fig. 6E,F), but also in the posterior region of the ventral foregut including the ventral pancreatic bud and the region where *Sox17* was intrinsically expressed (Fig. 6H; red arrowhead). These results suggest that STM loss results in ectopic activation of the liver program in the presumptive gall bladder and ventral pancreas regions. Previous studies have shown hepatic induction to occur in the ventral and dorsal foregut (Bossard and Zaret, 1998; Bossard and Zaret, 2000; Gualdi et al., 1996). During pancreas development, the FGF signaling pathway suppresses the liver program in the ventral and dorsal pancreas through an Fgf10/Fgfr2b/Sox9 pathway (Seymour et al., 2012). As *Fgf10* is expressed in the STM at E9.0 (Berg et al., 2007), this signaling pathway may suppress the liver program during the initial stages of gall bladder and ventral pancreas development. To examine when *Fgf10* was expressed in the STM, we examined the expression of the STM marker *Wt1* and of *Fgf10* before E9.0. At E8.5, *Fgf10* expression was not detected in the *Wt1*-expressing region (Fig. 6I,J; red arrowheads). At E9.0, *Fgf10* expression was observed in the *Wt1*-expressing STM near the ventral foregut (Fig. 6K,L), as previously reported. These results indicate that *Fgf10* expression in the STM begins at E9.0. At E9.25, *Fgf10* was expressed in the STM, and *Fgfr2b*, the main receptor for *Fgf10*, was expressed in the ventral foregut in the *Sox17*-expressing region (Fig. 6M–P). *Sox17* was not expressed in the *Alb+* presumptive liver region (Fig. 6Q,R), indicating that *Fgfr2b* is specifically expressed in the posterior region of the ventral foregut endoderm, that is, in the nascent gall bladder and ventral pancreas, but not in the nascent liver. These results indicate that after E9.0, the FGF signaling pathway functions specifically in the presumptive gall bladder and ventral pancreas regions through Fgf10/Fgfr2b. *Sox9*, a downstream target of Fgf10/Fgfr2b signaling (Seymour et al., 2012), was mainly expressed in the presumptive gall bladder and ventral pancreas regions, including the *Sox17*-expressing region (Fig. 6S,T). Thus, the FGF signaling pathway may function through Fgf10/Fgfr2b/Sox9 to suppress the liver program in the nascent gall bladder and ventral pancreas shortly after liver formation begins (Fig. 6U). At E9.25, the expression of *Fgf10* was lost near the ventral foregut in *Mab21l2*-deficient embryos (Fig. 6W; red arrowhead) compared to wild-type embryos (Fig. 6V), and *Sox9* expression was also significantly reduced in the ventral foregut endoderm (Fig. 6Y; red arrowhead) compared to wild-type embryos (Fig. 6X). These results suggest that after E9.0 (i.e., after hepatic induction), the STM suppresses ectopic activation of the liver program in the posterior region of the ventral foregut through the Fgf10/Fgfr2b/Sox9 signaling pathway.

## Discussion

### The STM is required for induction of gall bladder development

*Mab21l2* is expressed in the STM, but not in endothelial and ventral foregut endodermal cells. In *Mab21l2*-deficient embryos, the extrahepatic biliary system including the gall bladder was completely lost. At E9.0, the STM was completely absent near the posterior region of the ventral foregut, but normal endothelial cells were present. Previous studies have shown that the blood vessel endothelium plays an important role in the induction of pancreatic differentiation (Lammert et al., 2001) and that *Flk1*, which is expressed in endothelial cells adjacent to the liver bud, is required to promote liver bud growth (Matsumoto et al., 2001). These studies demonstrated that endothelial cells are essential for organogenesis of ventral foregut-derived tissues. However, the expression of *Flk1* was unchanged in *Mab21l2*-deficient embryos compared to wild-type embryos at E9.0, suggesting that the endothelial cells are normal. Thus, our results suggest that gall bladder loss resulted from loss of the STM, not from defects in the endothelial cells. Therefore, the STM is essential for the induction of gall bladder development.

### STM-derived BMP4 regulates differentiation of pancreatobiliary common progenitor cells into gall bladder progenitor cells by regulating *Sox17* and *Pdx1* expression

This study showed that *Sox17* and *Pdx1* were expressed in the same region, in the pancreatobiliary common progenitor cells, at E8.5, and that *Sox17*+/*Pdx1−* ventral foregut endodermal cells began to emerge at E9.0. These results indicate that after E9.0, the expression of *Sox17* and *Pdx1* in pancreatobiliary common progenitor cells is differentially regulated. Moreover, between E9.5 and E10.5, *Sox17* expression is specifically detected in the gall bladder primordium and *Pdx1* expression in the ventral pancreatic bud, showing that between E9.5 and E10.5 the differential regulation is complete. Our study also showed that after E9.0, the STM is adjacent only to the *Sox17*+, *Pdx1−* region, and is involved in the differentiation of pancreatobiliary common progenitor cells, most likely by maintaining *Sox17* expression and suppressing *Pdx1* expression. A previous study showed that the regulation of *Sox17* and *Pdx1* plays an important role in the differentiation of pancreatobiliary common progenitor cells into gall bladder progenitor cells and ventral pancreatic progenitor cells. *Sox17* is a master regulator of gall bladder formation (Spence et al., 2009). *Sox17* expression in *Mab21l2*-deficient embryos was reduced in the ventral foregut and *Pdx1* was ectopically expressed in the region where *Sox17* was intrinsically expressed, indicating that gall bladder loss in *Mab21l2*-deficient embryos resulted from defects in the regulation of *Sox17* and *Pdx1*. Thus, these results show that the STM is required to maintain *Sox17* expression and suppress *Pdx1* expression in the ventral foregut; the STM induces gall bladder development by differentially regulating *Sox17* and *Pdx1*.

Using whole-embryo cultures, we showed that Noggin reduced the expression of *Sox17* in the *Hhex*-positive foregut region of wild-type embryos, while *Sox17* expression in endothelial cells did not differ from expression in embryos cultured in control medium. This result suggests that Noggin specifically reduces *Sox17* expression in the foregut endodermal cells. Furthermore, *Hhex* expression in the foregut region was unchanged, suggesting that reduced expression of *Sox17* is unlikely to result from secondary effects of Noggin addition. Therefore, our results suggest that BMPs function to maintain *Sox17* expression in the ventral foregut. Several BMPs are expressed in the STM including BMP2, BMP4, BMP5, and BMP7 (Hogan, 1996; Zhao, 2003). Previous studies have shown that *Bmp4* is regulated by *FoxF1*, which is expressed in the STM and is important for gall bladder development (Kalinichenko et al., 2002; Mahlapuu et al., 2001). These observations suggest that *Bmp4* is involved in gall bladder development through regulation of *Sox17* and *Pdx1* expression. *Bmp4* was expressed in the STM at E8.5 when hepatic induction occurred (Rossi et al., 2001). However, in *Mab21l2*-deficient embryos at E9.0, STM loss resulted in the loss of *Bmp4* expression near the posterior region of the ventral foregut, including the presumptive gall bladder region, suggesting that reduced expression of *Sox17* may be a consequence of the loss of BMP4 signaling. BMP4 rescued *Sox17* expression in the foregut region of cultured *Mab21l2*-deficient embryos. A previous study showed that BMP4 promotes cell proliferation of *Sox17*-expressing cells (Sneddon et al., 2012). However, our study showed that Noggin did not influence the size of the *Sox17*-expressing region, suggesting that BMP4 is the primary regulator maintaining *Sox17* expression at this developmental stage. Moreover, BMP4 reduced *Pdx1* expression in the foregut of cultured wild-type embryos, suggesting that BMP4 also suppresses *Pdx1* expression. Therefore, our results demonstrate that, during gall bladder development, STM-derived BMP4 induces differentiation of *Sox17*+/*Pdx1*+ pancreatobiliary common progenitor cells adjacent to the STM into *Sox17*+/*Pdx1−* gall bladder progenitor cells by maintaining *Sox17* expression and suppressing that of *Pdx1*. The positional relationship between the STM and the ventral foregut endoderm thus determines the boundary between the gall bladder and the ventral pancreas (supplementary material Fig. S5).

Previous studies have shown that *Hgf* is expressed in the STM surrounding the hepatoblasts (Schmidt et al., 1995), and that the HGF receptor *c-met* is expressed in hepatoblasts (Bladt et al., 1995). During liver development, the Hgf-c-met signaling pathway promotes cell proliferation and survival of hepatoblasts through pro-mitogenic and anti-apoptotic actions (Birchmeier et al., 2003; Schmidt et al., 1995), indicating that the STM is involved in liver differentiation and growth. At E9.5, *Hgf* was expressed in the STM surrounding the gall bladder primordium (supplementary material Fig. S4A,B), and *c-met* was expressed in the gall bladder primordium (supplementary material Fig. S4C,D), indicating that the STM promotes the proliferation and survival of gall bladder primordial cells. Therefore, the STM is involved in both differentiation and growth of the gall bladder.

### The STM suppresses ectopic activation of the liver program in the presumptive gall bladder and ventral pancreas

Previous studies in chick and *Xenopus* suggest that during endoderm patterning, a concentration gradient of Wnt and FGF secreted from the adjacent mesoderm regulates the regional identity of the endoderm (foregut, midgut, or hindgut) (Dessimoz et al., 2006; McLin et al., 2007; Wells and Melton, 2000). Moreover, inhibition of the Wnt signaling pathway in the *Xenopus* posterior endoderm induces ectopic activation of the liver program in the intestine (McLin et al., 2007). These studies indicate that for each organ to develop in the appropriate position, not only inductive signals but also suppressive signals are required. After endoderm patterning, FGF from the cardiac mesoderm induces or suppresses liver development in the foregut endoderm, depending on the region (Deutsch et al., 2001; Jung et al., 1999). In *Mab21l2*-deficient embryos, in which the STM was completely lost near the posterior region of the ventral foregut, the expression of *Alb* was activated not only in the nascent liver but also in the remaining posterior region of the ventral foregut, suggesting that after hepatic induction, the STM normally suppresses ectopic activation of the liver program in the presumptive gall bladder and ventral pancreas regions.

Various genes that encode secreted ligands, including *Fgf10*, are expressed in the STM. During pancreas development, *Fgf10* is expressed in the mesenchyme surrounding the pancreatic epithelium, and *Fgfr2b* and *Sox9* are expressed in the pancreatic epithelium. In addition, the Fgf10-Fgfr2b signaling pathway suppresses the liver program in the pancreatic epithelium by regulating *Sox9* (Seymour et al., 2012), suggesting that FGF10 from the STM has similar functions. Here, we showed that *Fgf10* is expressed in the STM at E9.0, and *Fgfr2b* and *Sox9* were expressed in presumptive gall bladder and ventral pancreas regions, and not in the nascent liver. This expression pattern enables the Fgf10/Fgfr2b/Sox9 signaling pathway to function in a region-specific manner in the presumptive gall bladder and ventral pancreas. In *Mab21l2*-deficient embryos, STM loss resulted in the loss of *Fgf10* expression near the ventral foregut. In addition, *Sox9* expression in the ventral foregut was significantly reduced. These results suggest that FGF10 from the STM is involved in suppression of the liver program through the coordinated expression of Fgf10/Fgfr2b/Sox9 in the presumptive gall bladder and ventral pancreas and that the resulting expression pattern determines the boundary between hepatic and pancreatobiliary common progenitors. Therefore, the STM functions not only in hepatic induction and liver growth, but also in suppression of the liver program in the nascent gall bladder and ventral pancreas (supplementary material Fig. S5).

Taken together, this study revealed that the establishment of an appropriate positional relationship between the STM and the ventral foregut endoderm is required for proper organogenesis in the ventral foregut, and that the STM induces the formation first of the liver and then of the gall bladder through the changes in the positional relationship of the STM to the ventral foregut at each developmental stage (supplementary material Fig. S5).

## Supplementary Material

Supplementary Material
