# Supplementary material for: The septum transversum mesenchyme induces gall bladder development
Source: Biol Open. 2013 Jun 20;2(8):779–88. doi: 10.1242/bio.20135348 (PMC3744069; doi:10.1242/bio.20135348)
Supplement: Supplementary Material [file supp_2_8_779__index.html]

The septum transversum mesenchyme induces gall bladder development — The septum transversum mesenchyme induces gall bladder development — Supplementary Material 

# The septum transversum mesenchyme induces gall bladder development

## 

**Files in this Data Supplement:**

- Supplementary Material - Yohei Saito et al. doi: 10.1242/bio.20135348
