# Supplementary material for: The septum transversum mesenchyme induces gall bladder development
Source: Biol Open. 2013 Jun 20;2(8):779–88. doi: 10.1242/bio.20135348 (PMC3744069; doi:10.1242/bio.20135348)
Supplement: Supplementary Material [file supp_bio.20135348_bio.20135348-s1.pdf]

## Supplementary Material

**Yohei Saito et al. doi: 10.1242/bio.20135348**

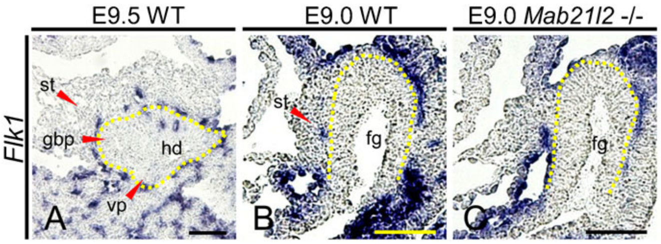


**Fig. S1. The expression pattern of *Flk1*.** *In situ* hybridization for the indicated transcripts was conducted using embryo paraffin sections. (**A**) Sagittal section of an E9.5 wild-type embryo showing that *Flk1* was expressed in endothelial cells surrounding the gall bladder primordium and ventral pancreatic bud. (**B**,**C**) Sagittal sections of wild-type and *Mab21l2*-deficient embryos showing that at E9.0, the expression of *Flk1* was unchanged in *Mab21l2*-deficient embryos compared to wild-type embryos. st, septum transversum mesenchyme; fg, foregut; gbp, gall bladder primordium; vp, ventral pancreatic bud; hd, hepatic diverticulum. Scale bars: 30 µm.

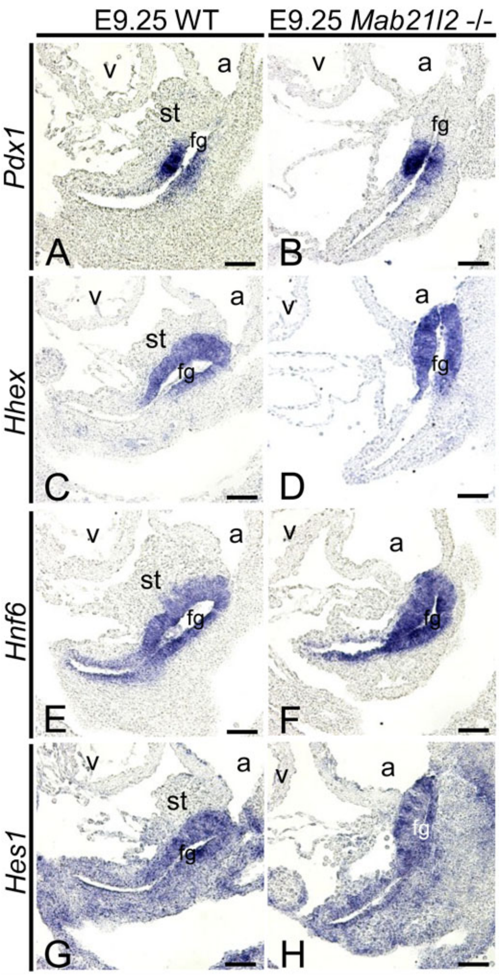


**Fig. S3. Expression of genes required for gall bladder and pancreas development.** (**A–H**) *In situ* hybridization for the indicated transcripts was conducted using embryo paraffin sections. The expression of *Pdx1*, *Hhex*, *Hnf6*, and *Hes1* was unchanged in *Mab21l2*-deficient embryos compared to wild-type embryos. a, atrium; v, ventricle; st, septum transversum mesenchyme; fg, foregut. Scale bars: 30 µm.

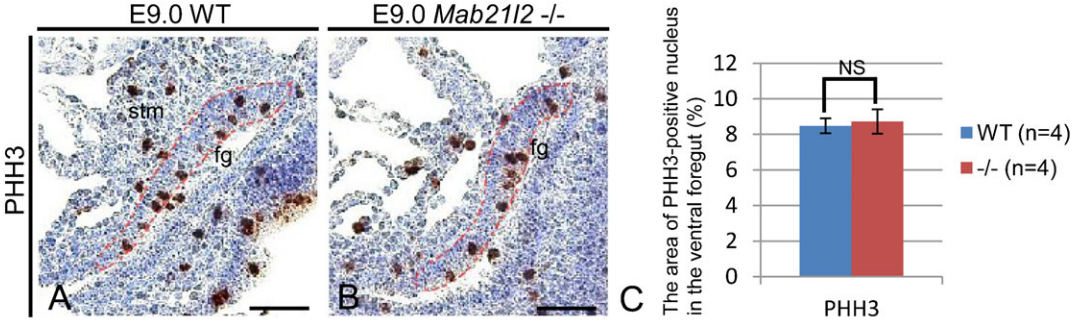


**Fig. S2. Proliferation of ventral foregut endodermal cells.** (**A**,**B**) Immunohistochemical staining of phosphorylated histone H3 (PHH3) in paraffin sections shows that proliferation of ventral foregut endodermal cells was unchanged in E9.0 *Mab21l2*-deficient embryos compared to wild-type embryos. (**C**) Quantification of proliferating ventral foregut endodermal cells. The values show the means of the percentage of the ventral foregut area (red dashed lines) occupied by PHH3-positive nuclei. Error bars represent the standard deviation (WT, $n$=4; *Mab21l2* (−/−), $n$=4) and $P$ values were calculated using the two-tailed Student's $t$-test. Scale bars: 30 µm.

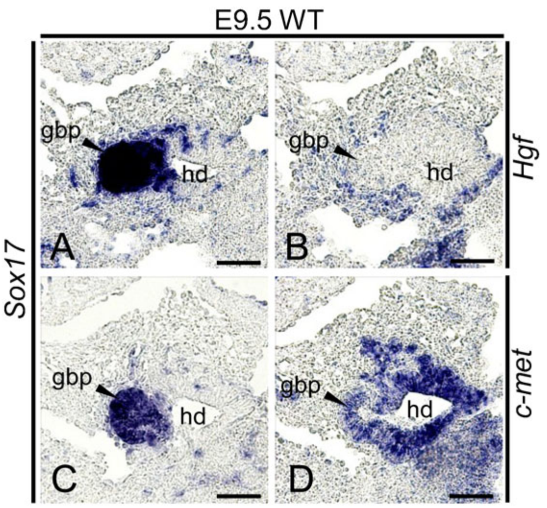


**Fig. S4. Expression pattern of *Hgf* and *c-met*.** *In situ* hybridization for the indicated transcripts was conducted using embryo paraffin sections. Sagittal serial sections of an E9.5 wild-type embryo showing that *Hgf* was expressed in the STM surrounding the *Sox17*-positive gall bladder primordium (**A**,**B**), and *c-met* was expressed in the *Sox17*-positive gall bladder primordium (**C**,**D**). gbp, gall bladder primordium; hd, hepatic diverticulum. Scale bars: 30 μm.

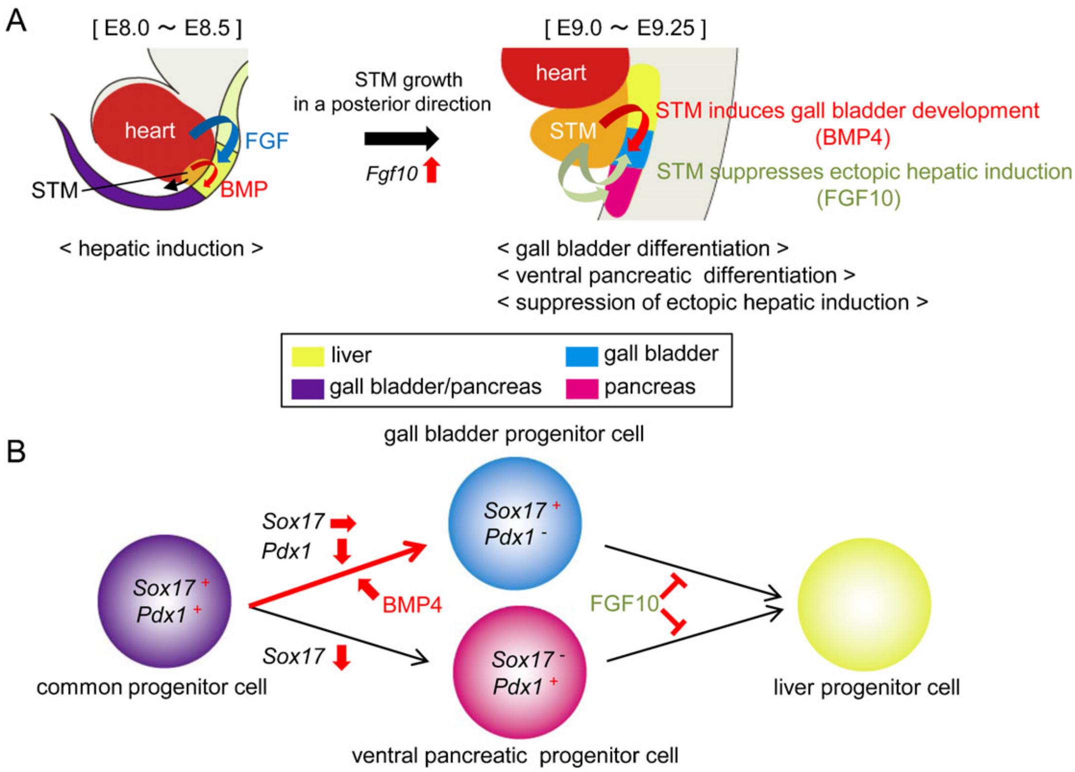


**Fig. S5. A model of ventral foregut-derived organogenesis.** (**A**) Hepatic induction occurs from E8.0 to E8.5 in the ventral foregut due to the production of FGF by the cardiac mesoderm and BMP by the STM. The ventral foregut endodermal progenitor cells differentiate into liver progenitor and pancreatobiliary common progenitor cells. From E8.5 to E9.0, the STM grows in a posterior direction, becoming adjacent to the presumptive gall bladder region, but not to the presumptive ventral pancreas region. From E9.0 to E9.25, the STM induces gall bladder development in a region-specific manner through the action of BMP4. Furthermore, *Fgf10* is upregulated in the STM from E8.5 to E9.0, and production of FGF10 suppresses the ectopic activation of the liver program in the presumptive gall bladder and ventral pancreatic region. Thus, the STM plays pivotal roles in posterior ventral foregut-derived organogenesis by controlling differentiation. (**B**) During the differentiation process in the posterior region of the ventral foregut, *Sox17*+/*Pdx1*+ pancreatobiliary common progenitor cells differentiate into *Sox17*+/*Pdx1*− gall bladder progenitor cells in response to BMP4 from the STM. In the absence of BMP4, common progenitor cells downregulate *Sox17* and differentiate into *Sox17*−/*Pdx1*+ ventral pancreatic progenitor cells. During this process, the liver program is suppressed in the gall bladder and in ventral pancreatic progenitor cells by the action of FGF10 produced by the STM. Thus, during the induction of gall bladder and ventral pancreas development, STM-derived signals control differentiation of the progenitor cells of each organ.

Biology Open
